# Beyond one‐size‐fits‐all: Reimagining well‐being programmes in medical education through student expectations and agency

**DOI:** 10.1111/medu.15604

**Published:** 2024-12-24

**Authors:** Nabeela Kajee, Elize Archer

**Affiliations:** ^1^ Department of Psychiatry University of Oxford, Warneford Hospital Oxford UK; ^2^ Department of Health Professions Education (DHPE), Faculty of Medicine and Health Sciences (FMHS) University of Stellenbosch Cape Town South Africa

## Abstract

Kajee & Archer comment on Tan et al.'s exploration of student well‐being programs, outlining additional implications for medical schools.

In this issue of the journal, Tan et al.^1^ offer an excellent contribution to the study of medical education through their paper entitled ‘How Do Medical Students' Expectations Shape Their Experiences of Wellbeing Programs?’^1^ The authors explore an important gap in the literature by asking how individual medical students' personal backgrounds relate to their experiences of the well‐being programmes offered by their institution. Through a qualitative case study methodology and an ‘Institutional Ethnographic’ lens, semi‐structured interviews were conducted with medical students. Further, document analysis was conducted, and field notes were analysed alongside the interviews.

The article contextualises the school's well‐being programme by explaining it to be mandatory, offered through a ‘House System’, and involving the assignment of personal tutors to students. In parallel, partially funded by the medical school in an unofficial capacity, runs the ‘HouseFam’ system of student‐for‐student well‐being support. Tan et al.^1^ explored how students make meaning from these systems by focusing on promises, sense‐making, reciprocity, empowerment and legitimacy. The contrast between the two systems is particularly compelling and informative.

The well‐being supports offered through the institutionally‐run House System appear to play a useful role in forming friendships, building peer support and implementing events such as the White Coat Ceremony. It appears, in other words, to be a valuable resource, while at the same time, raising important questions about the extent to which some medical students can relate to formalised programmes. The experiences of those who ‘felt like outsiders’ is of particular interest in this regard as they appeared more likely to have unmet ‘expectations’. It is unfortunately ironic that even our wellness programmes can leave people feeling ‘othered’ or ‘excluded’. Further research is needed to understand the factors that cause such feelings and the relationship between intersectionality, bias and prejudice.^2^, ^3^ Tan et al. offer an intriguing lead though when they observe that some students felt their interactions with well‐being tutors to be ‘transactional’ rather than ‘relational’ in nature.

It is unfortunately ironic that even our wellness programmes can leave people feeling “othered” or “excluded.”

In hindsight, we should likely not be surprised that reciprocity between student and tutor is ‘continuously contested’ even within the social contract intended to enable wellness. Any programme that does not actively include its participants in every stage of planning, development and quality improvement is likely to struggle to create an engaged and embodied student user‐base. That the medical school indirectly supports the parallel programme established by the medical students suggests an institution that is very much working towards supporting well‐being among its students; that students felt compelled to establish a secondary programme of their own, however, is strongly reminiscent of the ‘nothing for us without us’ mantra. It will be intriguing to see if future research can address further questions regarding the power‐positioning of different types of support structures and whether the student‐led one being ‘alternative’ is, in and of itself, influential. To what extent is activism aimed at being inclusive particularly well‐received by virtue of it being seen to a degree as subversive?

Any programme that does not actively include its participants in every stage of planning, development and quality improvement is likely to struggle to create an engaged and embodied student user‐base.

As the literature suggests, medical student expectations are a complex phenomenon; both in content and context.^4^ That the best of intentions can yield variable and unexpected reactions is all the more reason for ‘co‐creation’ and personalised streams within well‐being programmes. The importance and timeliness of such is particularly relevant when one recognises the increasing rates at which mental illness is impacting medical students in various settings.^5^, ^6^

In other words, as this study encourages us to widen conversations surrounding the experience‐expectation mismatch felt by some medical students in some well‐being programmes, it is time to consider if we could change the ideological approach to well‐being by recentring the individual student rather than assuming one‐size fits all. Just as one may expect to use many physical tools in a lifetime, so too, could many well‐being approaches offer different value at different points in time or to different people. The student ‘agency’ outlined in Tan's work does not invalidate or even reduce the importance of institutionally driven programmes, but it does suggest that peer‐peer learning and senior mentorship/support may each have a place and need to be used thoughtfully and flexibly.

The student ‘agency’ outlined in Tan's work does not invalidate or even reduce the importance of institutionally driven programmes, but it does suggest that peer‐peer learning and senior mentorship/support may each have a place and need to be used thoughtfully and flexibly.

Organisations can function with a ‘top‐down’ and ‘bottom‐up’ hybrid in a manner that enables idea exchange and power‐sharing.^7^ Traditional unidirectional, unilateral decisions are by nature biased and exclusionary given the siloed nature of concept generation and execution. Dynamic exchanges of ideas in the well‐being space may lead to more responsive and generative approaches. To that end, we also encourage exploration of generational and gender differences, important parts of the conversation surrounding well‐being programmes that were not clearly explored by this study.

In sum, Tan et al. lay a valuable foundation for future research into structuring and supporting well‐being programmes in medical schools. They reinforce that well‐being programmes play a crucial role in fostering healthy medical students and medical school climates. Hierarchically enforced well‐being programmes, however, are called into question, as students were reported to democratise meaning‐structures through adaptation and building secondary spaces. As such, the findings encourage medical schools and stakeholders to critically reflect on the individual experiences of their own medical students and, in doing so, to consider their mandate to offer equitable, accessible and universally engaging well‐being programmes to their students.

Hierarchically enforced well‐being programmes, however, are called into question, as students were reported to democratise meaning‐structures through adaptation and building secondary spaces.

## AUTHOR CONTRIBUTIONS

Nabeela Kajee and Elize Archer conceived the commentary. Nabeela Kajee drafted the commentary. Nabeela Kajee and Elize Archer revised the developing commentary and approved the final version.

## Data Availability

Data sharing not applicable to this article as no datasets were generated or analysed during the current study.
